# Anti-High Mobility Group Box-1 Monoclonal Antibody Attenuates Seizure-Induced Cognitive Decline by Suppressing Neuroinflammation in an Adult Zebrafish Model

**DOI:** 10.3389/fphar.2020.613009

**Published:** 2021-03-01

**Authors:** Yam Nath Paudel, Iekhsan Othman, Mohd. Farooq Shaikh

**Affiliations:** ^1^ Neuropharmacology Research Strength, Jeffrey Cheah School of Medicine and Health Sciences, Monash University Malaysia, Bandar Sunway, Malaysia; ^2^ Liquid Chromatography-Mass Spectrometry Platform, Jeffrey Cheah School of Medicine and Health Sciences, Monash University Malaysia, Bandar Sunway, Malaysia

**Keywords:** anti-HMGB1 mAb, epilepsy, neuroinflammation, seizures, cognitive decline, zebrafish

## Abstract

Epilepsy is a chronic brain disease afflicting around 70 million global population and is characterized by persisting predisposition to generate epileptic seizures. The precise understanding of the etiopathology of seizure generation is still elusive, however, brain inflammation is considered as a major contributor to epileptogenesis. HMGB1 protein being an initiator and crucial contributor of inflammation is known to contribute significantly to seizure generation via activating its principal receptors namely RAGE and TLR4 reflecting a potential therapeutic target. Herein, we evaluated an anti-seizure and memory ameliorating potential of an anti-HMGB1 monoclonal antibody (mAb) (1, 2.5 and 5 mg/kg, I.P.) in a second hit Pentylenetetrazol (PTZ) (80 mg/kg, I.P.) induced seizure model earlier stimulated with Pilocarpine (400 mg/kg, I.P.) in adult zebrafish. Pre-treatment with anti-HMGB1 mAb dose-dependently lowered the second hit PTZ-induced seizure but does not alter the disease progression. Moreover, anti-HMGB1 mAb also attenuated the second hit Pentylenetetrazol induced memory impairment in adult zebrafish as evidenced by an increased inflection ration at 3 and 24 h trail in T-maze test. Besides, decreased level of GABA and an upregulated Glutamate level was observed in the second hit PTZ induced group, which was modulated by pre-treatment with anti-HMGB1 mAb. Inflammatory responses occurred during the progression of seizures as evidenced by upregulated mRNA expression of HMGB1, TLR4, NF-κB, and TNF-α, in a second hit PTZ group, which was in-turn downregulated upon pre-treatment with anti-HMGB1 mAb reflecting its anti-inflammatory potential. Anti-HMGB1 mAb modulates second hit PTZ induced changes in mRNA expression of CREB-1 and NPY. Our findings indicates anti-HMGB1 mAb attenuates second hit PTZ-induced seizures, ameliorates related memory impairment, and downregulates the seizure induced upregulation of inflammatory markers to possibly protect the zebrafish from the incidence of further seizures through via modulation of neuroinflammatory pathway.

## Introduction

Epilepsy is a devastating neurological disease characterized by a rapid enduring tendency to generate epileptic seizures that might be due to abnormal brain activity. Epilepsy is a serious health concern distressing about 70 million global population (Thijs et al., 2019). Despite the recent advances in clinical and pre-clinical epilepsy research, people at the risk of epilepsy can be easily identified but cannot modulate disease progression due to non-availability of disease-modifying anti-epileptic drugs (AEDs) (Dichter, 2009). Although the etiopathogenesis of epilepsy is not yet completely understood, several causative factors are speculated to play a role. They include brain injuries (neurotrauma, stroke, brain tumors), mutations of specific genes, infection of the central nervous system (CNS), metabolic disorders and autoimmune disorders (Scheffer et al., 2017). The precise understanding of the pathogenesis of epileptogenesis and seizure recurrence remains the topic of tremendous pre-clinical and clinical investigation and their identification would pave the way for the development of novel treatment strategies that not only halt the progression of the seizure but also minimizes the seizure burden (Vezzani et al., 2019). Despite the clinical availability of more than 25 AEDs that provides symptomatic relief by different mechanisms, more than 33% of epileptic patients suffer from the pharmacoresistant epilepsy (Kwan and Brodie, 2000). This reflects an immediate need for developing novel treatment approaches that could halt the disease progression and prevent seizure-associated neurobehavioral comorbidities.

High mobility group box 1 (HMGB1) is a DNA binding protein with molecular mass of 25-kDa and serve as a typical alarmin and a damage associated molecular patterns (DAMPs) (Harris et al., 2012). HMGB1 is a ubiquitous protein that has been reported to promote inflammation when released into the extracellular space post cellular activation, stress, damage, and death (Andersson et al., 2018). HMGB1 exerts its biological activity through the activation of its dominant receptors namely the receptor for advanced glycation end products (RAGE) and toll-like receptor 4 (TLR4) (Paudel et al., 2018a). The biological activity of HMGB1 depends on the location, nature of binding partners, and its redox states (Andersson et al., 2014; Tang et al., 2016). Structural elucidation of HMGB1 revealed it as a ubiquitous, highly conserved nuclear protein comprising of 215 amino acid residues. Furthermore, HMGB1 possess two DNA-binding terminals (termed A and B-boxes) and a negatively charged C-terminal tail that contains a recurring chain of 30 glutamic and aspartic acids (Aucott et al., 2018; Paudel et al., 2019a). In recent days, HMGB1 has been extensively explored in several HMGB1 mediated neurological pathologies including Epileptogenesis (Maroso et al., 2010; Paudel et al., 2019b), Parkinson’s disease (PD) (Gao et al., 2011; Angelopoulou et al., 2018), Alzheimer’s disease (AD) (Takata et al., 2004; Paudel et al., 2020a), Multiple sclerosis (MS) (Andersson et al., 2008; Paudel et al., 2019a) and ALS (Brambilla et al., 2018; Paudel et al., 2020c) and Brain injuries (Okuma et al., 2012; Paudel et al., 2020b).

The very first pioneering investigation unraveled that HMGB1-TLR4 signaling axis might contribute to the generation of epileptic seizures. This statement is attributed to the fact that there was an increased level of HMGB1 and TLR4 in both experimental epilepsy and epileptic brain samples from the human (Maroso et al., 2010). Worth mentioning here is that, HMGB1 inhibitor (Box A) and TLR4 inhibitor (*Rhodobacter sphaeroides* LPS) demonstrated anti-convulsive effect that further strengthens the role of HMGB1 and TLR4 in the generating epileptic seizures (Maroso et al., 2010). Furthermore, this finding reflects that HMGB1 might reflect as an emerging extracellular target against epilepsy (Paudel et al., 2019b).

HMGB1 neutralization strategy in a diverse range of HMGB1-mediated pathologies such as Epilepsy (Fu et al., 2017), MS (Robinson et al., 2013), Traumatic brain injury (TBI) (Okuma et al., 2012) and cognitive decline (Hei et al., 2018; Okuma et al., 2019) has been well reported. The extensively used HMGB1 targeting strategy against epilepsy is anti-HMGB1 monoclonal antibody (mAb) (Fu et al., 2017; Zhao et al., 2017) and Glycyrrhizin (natural inhibitor of HMGB1) (Luo et al., 2014; González-Reyes et al., 2016; Li et al., 2019).

Epilepsy merely exists alone and is often associated with several neurobehavioral conditions such as memory impairment, anxiety disorder, depression, autism spectrum disorder and psychiatric disorders with severe impact on patients' quality of life (Paudel et al., 2018b). Memory impairment, especially spatial memory is the most common comorbidities of temporal lobe epilepsy (TLE) (Cho et al., 2015). Rodent model of epilepsy has demonstrated that memory impairment is correlated with neuronal injuries in TLE (Kotloski et al., 2002; Do Val-da Silva et al., 2016). Memory impairment has been reported in Pentylenetetrazol (PTZ) induced seizure model in adult zebrafish (Kundap et al., 2019a). T-Maze test has been used to assess the memory impairment in the zebrafish were the time needed for the zebrafish to reach the deeper chamber is termed as the transfer latency (TL), which is also expressed as inflection ratio (Kundap et al., 2019a).

Even though anti-HMGB1 mAb has anti-epileptic effect on rodent model of epileptic seizures, the anti-convulsive effect of anti-HMGB1 mAb and its underlying mechanism on zebrafish seizure model is not yet explored. Hence the current investigation aimed to evaluate the anti-epileptic and memory ameliorating effect of anti-HMGB1 mAb in a second hit PTZ induced seizure model in adult zebrafish.

## Materials and Methods

### Chemicals and Experimental Equipment

All the reagents used are of analytical grade. Pilocarpine, PTZ, Glutamate, γ-aminobutyric acid (GABA), DZP, and Anti-HMGB1 mAb (anti-body purified in mouse, cline 2F6, purified immunoglobulin, WH0003146M8) were purchased from Sigma Aldrich (United States). Dimethyl sulfoxide (DMSO) was purchased from Vivantis Inc. (United States). TRIzol® reagent was purchased from Invitrogen, Carlsbad, CA, United States. Ethanol 95% (EtOH) was purchased from Kolin Chemicals Co. Ltd., Korea, methanol (MeOH), chloroform (CHCl3), isopropanol (IPA), and formic acid was purchased from Friedemann Schmidt Chemicals, Parkwood 6147, Western Australia. The MilliQ system from Millipore (Bedford, MA, United States) was used to produce pure water when needed. Agilent 1290 Infinity UHPLC, coupled with the Agilent 6410 Triple, Quad LC/MS was used to quantify the brain neurotransmitter levels. Gene expression study was performed with the Applied Biosystems StepOnePlus™ Real-Time PCR System.

### Software

The Smart V3.0.05 tracking software (Pan Lab, Harvard Apparatus) was used for the tracking of zebrafish swimming patterns and locomotion parameters (Seizure behavior and T-maze tracking). All the videos (Seizure behavior and T-maze tracking) were recorded by Sony HDR-PJ340E video camera (recording at 50 frames per second).

### Animals

Adult zebrafish (*Danio rerio*) of 3–4 months (wild-type strain) were purchased from University Putra Malaysia (UPM), Malaysia. All animals were held at the standard husbandry conditions in the animal facilities of Monash University Malaysia. The water for zebrafish tank is temperature maintained (between 26°C and 30°C), pH maintained (between pH 6.8 and pH 7.1) and kept at a 250-lx light intensity with a cycle of 14-h of light to 10 h of darkness. The lights of the zebrafish room were fixed with timer that automatically turns on and off the light at 8 am and 10 pm respectively. TetraMin^®^ Tropical Flakes in addition with live brine shrimps (artemia) procured from Bio-Marine (Aquafauna, Inc. United States) was used to feed the zebrafish (thrice a day). Zebrafish were housed in a standard zebrafish tanks (length 36 cm; a width 26 cm; and a height of 22 cm) and connected with a water circulation system to provide a constant aeration. All animal experimentation was authorized and approved by the Animal Ethics Committee of Monash University Malaysia.

### Second Hit PTZ (Prior Stimulated With Pilocarpine) Seizure Model

The main rationale behind the second hit PTZ model is to develop the chronic seizure-like condition in the adult zebrafish. Our protocol spans from day 1 to day 10 where sub-convulsive dose of PTZ (80 mg/kg, I.P.) is injected at day 10 to the epileptic zebrafish that was prior stimulated with Pilocarpine (400 mg/kg, I.P.) at day 1, hence named as the second hit PTZ seizure model.

Pilocarpine and PTZ administered zebrafish demonstrate diverse seizure-like behaviors with difference in seizures intensities and latency and achieving the different seizure scores. PTZ induced seizure-like behaviors will last for about 15 min post-PTZ administration and steadily normalize with time. Pro-convulsants (Pilocarpine and PTZ) were injected I.P. rather than dissolving in the water as per earlier reported study (Mussulini et al., 2013). This is because little volume of water is used to restrict the number of pro-convulsants required and having the zebrafish in such a small water volume might impact on the learning and memory. The PTZ induced zebrafish at day 10 were then shifted to a 13-L observation tank filled three-quarters of the way with water. The behavior of the zebrafish was then recorded for 15 min after recovery from anesthesia and the video was later viewed to quantify the seizure score. The highest seizure score was the highest noted seizure score for the entire 15 min duration of the seizure recording.

The dose of Pilocarpine in adult zebrafish are based on our prior study (Paudel et al., 2020e). Besides, the seizure score for Pilocarpine-induced seizure has been determined by evaluating the swimming pattern of epileptic zebrafish as per our earlier study (Paudel et al., 2020e) (Table 1). The sub-convulsive dose of PTZ (80 mg/kg, I.P.) was selected from earlier study (Kundap et al., 2019b), and PTZ induced seizure score in adult zebrafish was recorded as per the prior documented scoring system (Kundap et al., 2017) (Table 2).

**TABLE 1:** Pilocarpine induced seizure scoring system.

Score	Criteria
Score 0	Normal swimming
Score 1	Jittery movement at the top of the tank
Score 2	Ataxia/Hyperactivity
Score 3	Circular movement, circling around small area
Score 4	Erratic burst movement with loss of posture/Corkscrew swimming

**TABLE 2:** PTZ induced seizure scoring system.

Score	Criteria
Score 1	Short swim mainly at the bottom of the tank.
Score 2	Increased swimming activity and high frequency of opercula movement.
Score 3	Burst swimming, left and right movements as well as the erratic movements.
Score 4	Circular movements.

### Drug Treatments and Experimental Groups

**FIGURE 1:**
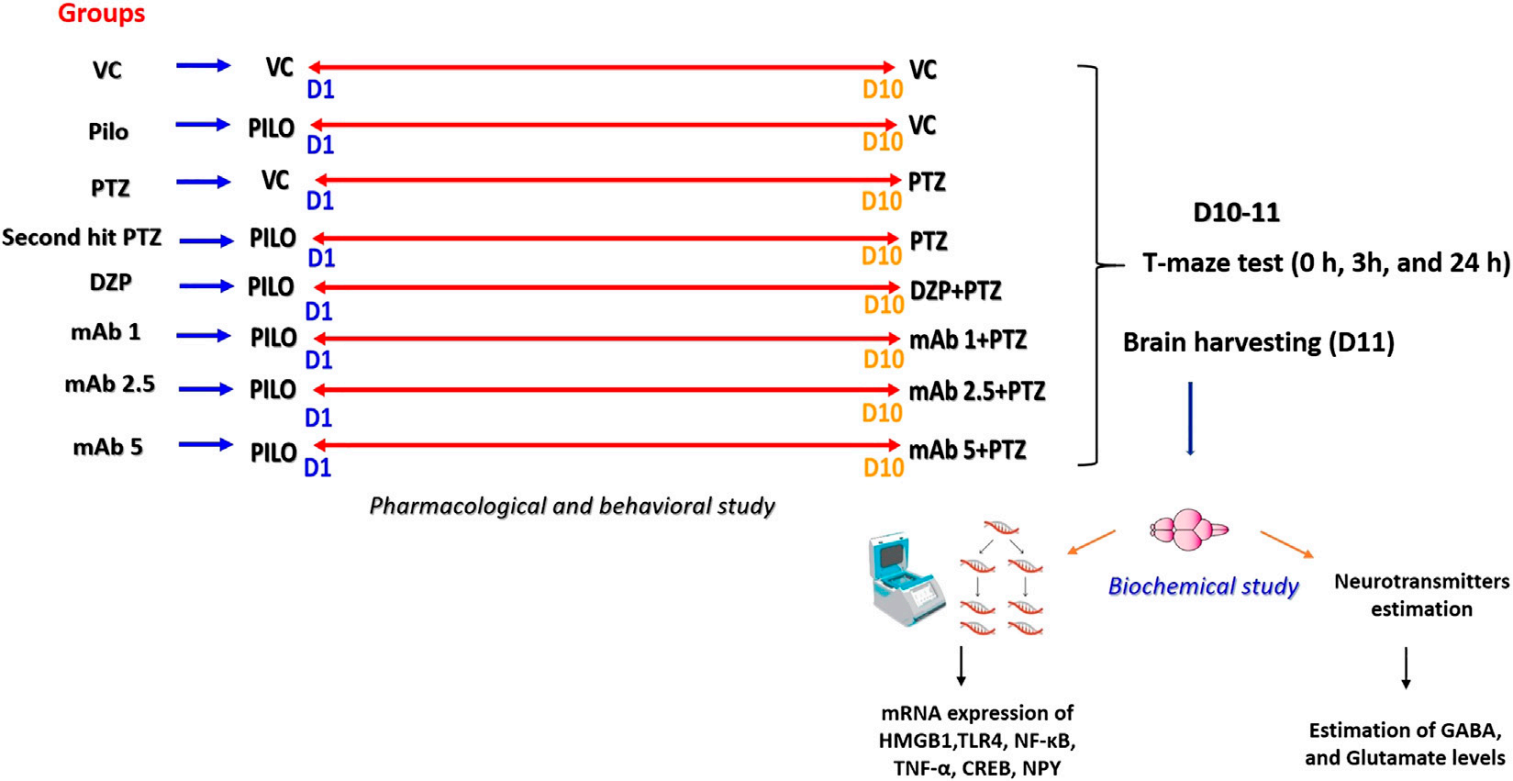
Pictorial representation of experimental procedure. The figure depicts the several time points (at day 1 and day 10) in the corresponding experimental groups where treatments were performed with VC, Pilo, PTZ, DZP and three doses of mAb. In day 1 and day 10, there is a recording of locomotor behavior for 15 min for each experimental group. On day 10, in the DZP groups and mAbs groups, pre-treatment was done 30 min prior to PTZ administration. Starting from day 10, T-maze test were performed for each group at the interval of 0h, 3h and 24 h. After 24 h T-maze test, all the fish were euthanized, and brain samples were collected which were further subjected to biochemical investigations. D1, Day 1; D10, Day 10, D11, Day 11; VC, Vehicle control (10% DMSO, I.P.); DMSO, dimethyl sulfoxide; I.P., Intraperitoneal; Pilo, Pilocarpine (400 mg/kg, I.P.); PTZ, Pentylenetetrazol (80 mg/kg, I.P.); DZP, Diazepam (1.25 mg/kg, I.P.); mAb 1, Anti-HMGB1 mAb (1 mg/kg, I.P.); mAb 2.5, Anti-HMGB1 mAb (2.5 mg/kg, I.P.); mAb 5, Anti-HMGB1 mAb (5 mg/kg, I.P.).

The adult zebrafish (n = 12) is divided into eight corresponding groups and precise details of the entire experimental groups is shown in Table 3. The experimental groups VC, Pilo, and PTZ is to ensure the validity of second hit PTZ group. The remaining pre-treated groups namely DZP, mAb1, mAb 2.5, and mAb 5 respectively were injected with Pilocarpine in day 1 and PTZ in day 10. Pre-treatments with DZP, mAb 1, mAb 2.5, and mAb 5 were done 30 min prior to PTZ injection in day 10 (Figure 1) (Table 3).

**TABLE 3:** Experimental groups evaluating therapeutic potential of anti-HMGB1 mAb against second hit PTZ induced cognitive Decline.

Experimental groups	Description
VC	VC at Day 1 and Day 10
Pilo	Pilo at Day 1 + VC at Day 10
PTZ	VC at Day 1 + PTZ at Day 10
Second hit PTZ	Pilo at Day 1 + PTZ at Day 10
DZP	Pilo at Day 1 + Pre-treatment with DZP and PTZ at Day 10
mAb 1	Pilo at Day 1+ Pre-treatment with mAb 1 and PTZ at Day 10
mAb 2.5	Pilo at Day 1+ Pre-treatment with mAb 2.5 and PTZ at Day 10
mAb 5	Pilo at Day 1+ Pre-treatment with mAb 5 and PTZ at Day 10

VC, Vehicle control (10% DMSO, I.P.); DMSO, dimethyl sulfoxide; I.P., Intraperitoneal; Pilo, Pilocarpine (400 mg/kg, I.P.); PTZ, Pentylenetetrazol (80 mg/kg, I.P.); DZP, Diazepam (1.25 mg/kg, I.P.); mAb 1, Anti-HMGB1 mAb (1 mg/kg, I.P.); mAb 2.5, Anti-HMGB1 mAb (2.5 mg/kg, I.P.); mAb 5, Anti-HMGB1 mAb (5 mg/kg, I.P.).

The dose of mAb was modified from prior published study (Fu et al., 2017; Zhao et al., 2017). mAb is dissolved in 2% phosphate buffer saline (PBS). Before each I.P. injection, the zebrafish were individually immersed in an anesthesia solution (30 mg/L benzocaine) until the termination of the movement.

Brain samples from all the groups are harvested at day 11 (after 24 h T-Maze test) and proceeds for biochemical analysis (gene expression and neurotransmitters estimation). Experimental workflow detailing all the experimental groups and procedure is shown in (Figure 1).

### T-Maze Test for Learning and Memory

**FIGURE 2:**
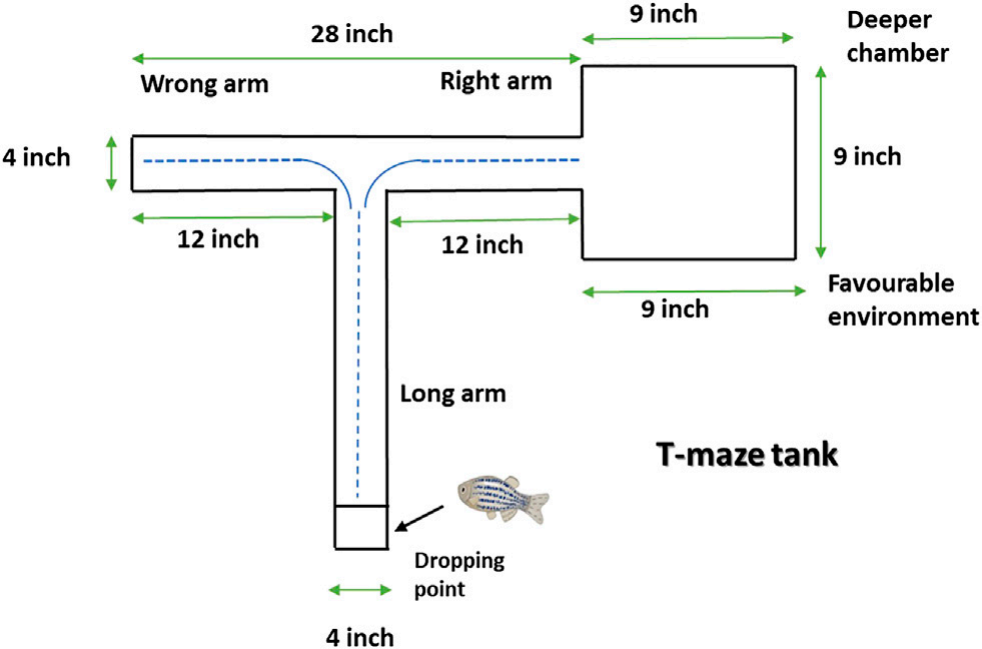
Representative diagram of T-maze tank. Zebrafish is kept at the dropping point and recorded the time taken to reach the deeper/bigger chamber. The blue dotted line denotes the representative swimming pattern of zebrafish that begins from the dropping point.

T-maze entails one long (45.72 cm) and two shorts (30.48 cm) arm whereby one short arm is tied to the deeper square chamber (22.86 cm × 22.86 cm) (Figure 2). The deeper chamber environment is favored by zebrafish. Once the zebrafish reaches the deeper chamber, they remain there due to the fact that the deeper chamber is deeper and wider as compared to the other T-maze arms (Kundap et al., 2017). The T-maze behavior test was performed in a behavior room, which was also kept at a temperature of between 26°C and 30°C and humidity between 50% and 60%. To begin with the T-maze test, zebrafish was placed into the beginning of the long arm and the zebrafish exploratory time was recorded for 5 min. TL in simple term is the time taken by the zebrafish to get to the deeper chamber. The T-maze test was conducted at an interval of 0, 3 and 24 h after second hit PTZ injection on day 10. The TL was stated as an inflection ratio (IR) whereby IR at 3 h post-challenge is calculated as (L0-L3)/(L3) and IR at 24 h post-challenge (IR24) calculated as (L0-L24)/(L24). Worth mentioning here is that L0, L3 and L24 represent the TL at 0, 3 and 24 h post-challenge, respectively.

### Brain Harvesting

At the end of the behavioral study (day 11), the zebrafish brains from the experimental group were harvested via eliminating the zebrafish skull and extracting the brain. All the harvested brains were shifted into the vial containing 200 μl of ice-cold methanol (1% formic acid) for neurotransmitter estimation studies whereas harvested brains were dissolved in the TRIzol® for the gene expression studies. All those harvested brains were stored immediately at −80°C till required.

### Brain Neurotransmitter Levels Study

Brain neurotransmitters (GABA and Glutamate) level was quantified using LC-MS/MS. The Stock solutions of the GABA and Glutamate were prepared in methanol (0.1% formic acid) and final concentration of 1 mg/ml were made and kept at 4°C until needed. A dilution range of 6.25–2,000 ng/ml were used for the calibration. Zebrafish’s brain from each experimental group was first homogenized in 200 μl of ice-cold methanol (1% formic acid). The brain homogenate was then vortex-mixed for 1 min and further centrifuged at 18,000 ×g for 10 min at 4°C. After All, the obtained supernatant was transferred into vials for the LC-MS/MS analysis. The details of the LC-MS/MS instrumentations were similar as reported in the earlier reported study from our laboratory (Kundap et al., 2019b).

### Gene Expression Study

#### RNA Isolation and Synthesis of First-Strand cDNA

The mRNA from the zebrafish brains from experimental groups was isolated according to the protocol supplied by the kit's manufacturer. The detailed protocol for the RNA isolation and synthesis of first stranded cDNA was performed as reported in our earlier study (Paudel et al., 2020e).

#### StepOne® Real-Time PCR

Gene expression of several genes (HMGB1, TLR4, TNF-α, NF-κB, CREB, NPY) and the housekeeping gene elongation factor 1-alpha-1b (eef1a1b) were determined from the real-time quantitative RT-PCR (Applied Biosystems) together with QuantiTect SYBR Green dye and the appropriate Qiagen primer set for each gene. The detailed protocol is followed as per our earlier investigation (Paudel et al., 2020e). Finally, the relative expression level (fold change) of the genes were determined by normalizing the threshold cycle (Ct) values acquired from the genes of interest, against the Ct value of the eef1a1b housekeeping gene using the formula: 2 ∧ [Ct eef1a1b − Ct Gene of interest].

QuantiTect SYBR Green dye (Qiagen, Valencia, CA, United States) was used for the gene expression study together with the following primer sets:

HMGB1: Dr_hmgb1b_2_SG QuantiTect Primer Assay (Cat no: QT02088555).

TLR4: Dr_tlr4ba_va. 1_SG QuantiTect Primer Assay (Cat no: QT02198539).

TNF-α: Dr_tnf_1_SG QuantiTect Primer Assay (Cat no: QT02097655).

NF-κB: Dr_nfkb1_2_SG QuantiTect Primer Assay (Cat no: QT02498762).

CREB_1: Dr._CREB_1 bpa_1_SG QuantiTect Primer Assay (Cat no: QT02197503).

NPY: Dr_npy_1_SG QuantiTect Primer Assay (Cat no: QT02205763).

eef1a1b: Dr_eef1a1b_2_SG QuantiTect Primer Assay (Cat no: QT02042684).

### Statistical Analysis

All values were expressed as mean ± SEM. Data were analyzed using one-way analysis of variance (ANOVA), followed by Sidak’s multiple comparison test. The *p*-values, ****p < 0.0001, ***p < 0.001, **p < 0 0.01 and ^*^ *p* < 0.05 were considered statistically significant depending on the test statistic. VC, Pilo, PTZ group is compared with second hit PTZ group whereas DZP and anti-HMGB1 mAb treated groups were compared with second hit PTZ group.

## Results

### Locomotor Pattern

**FIGURE 3:**
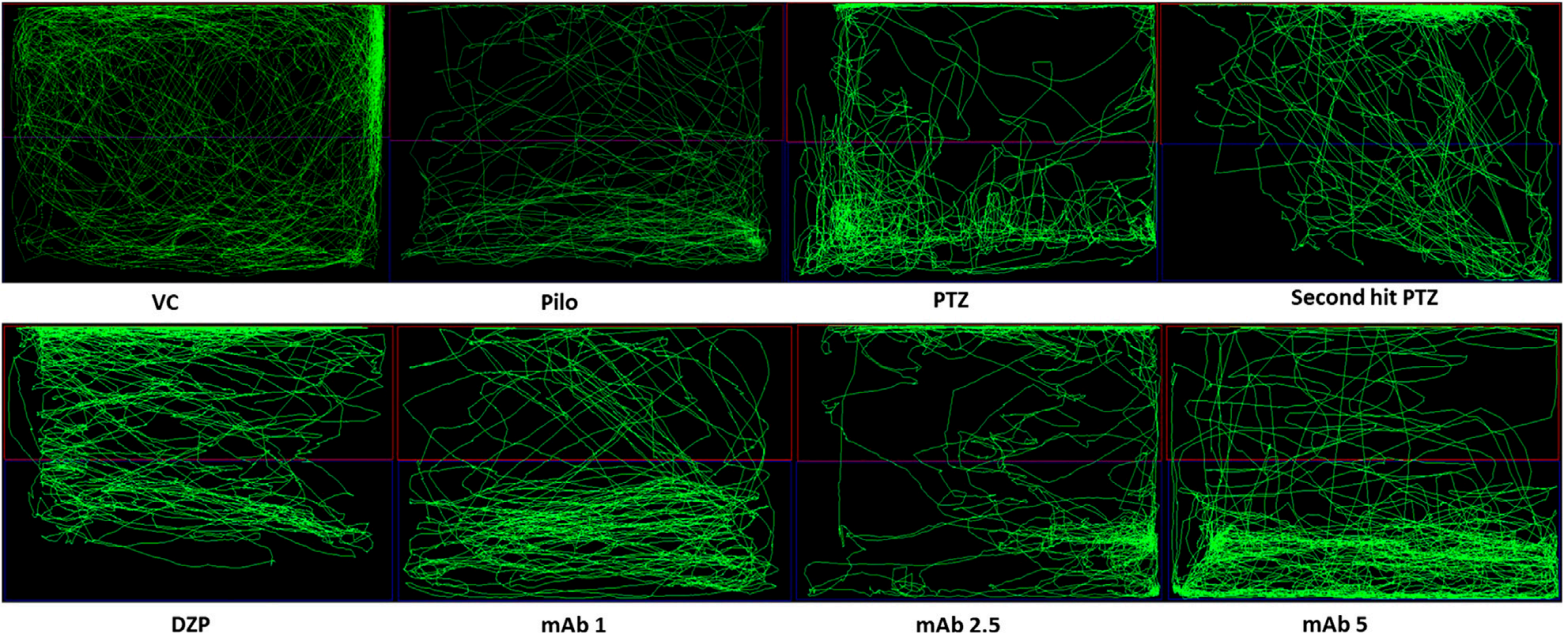
Representative swimming patterns for the corresponding experimental groups (n = 12) at day 10. Represents the swimming pattern of VC, Pilo, PTZ, second hit PTZ, DZP, mAb 1, mAb 2.5, and mAb 5 groups.

Abnormal locomotion is among the indicator of seizures in adult zebrafish. We assessed the effect of mAb on second hit PTZ induced behavioral disruption by evaluating the total distance traveled in the tank, time spend in upper and lower half of the tank, respectively. At day 10, normal swimming pattern was observed in the VC, Pilo and PTZ groups where the tracking pattern showed normal swimming throughout the tank (Figure 3). An involuntary, rapid body movement and hyperactivity was observed in the fish from second hit PTZ treated group and most swimming preference was for the upper half of the tank as shown in Figure 3. The swimming pattern of the fish from DZP, mAb 1, mAb 2.5, and mAb 5 pre-treated group demonstrated improved locomotor pattern with swimming throughout the tank (Figure 3).

**FIGURE 4:**
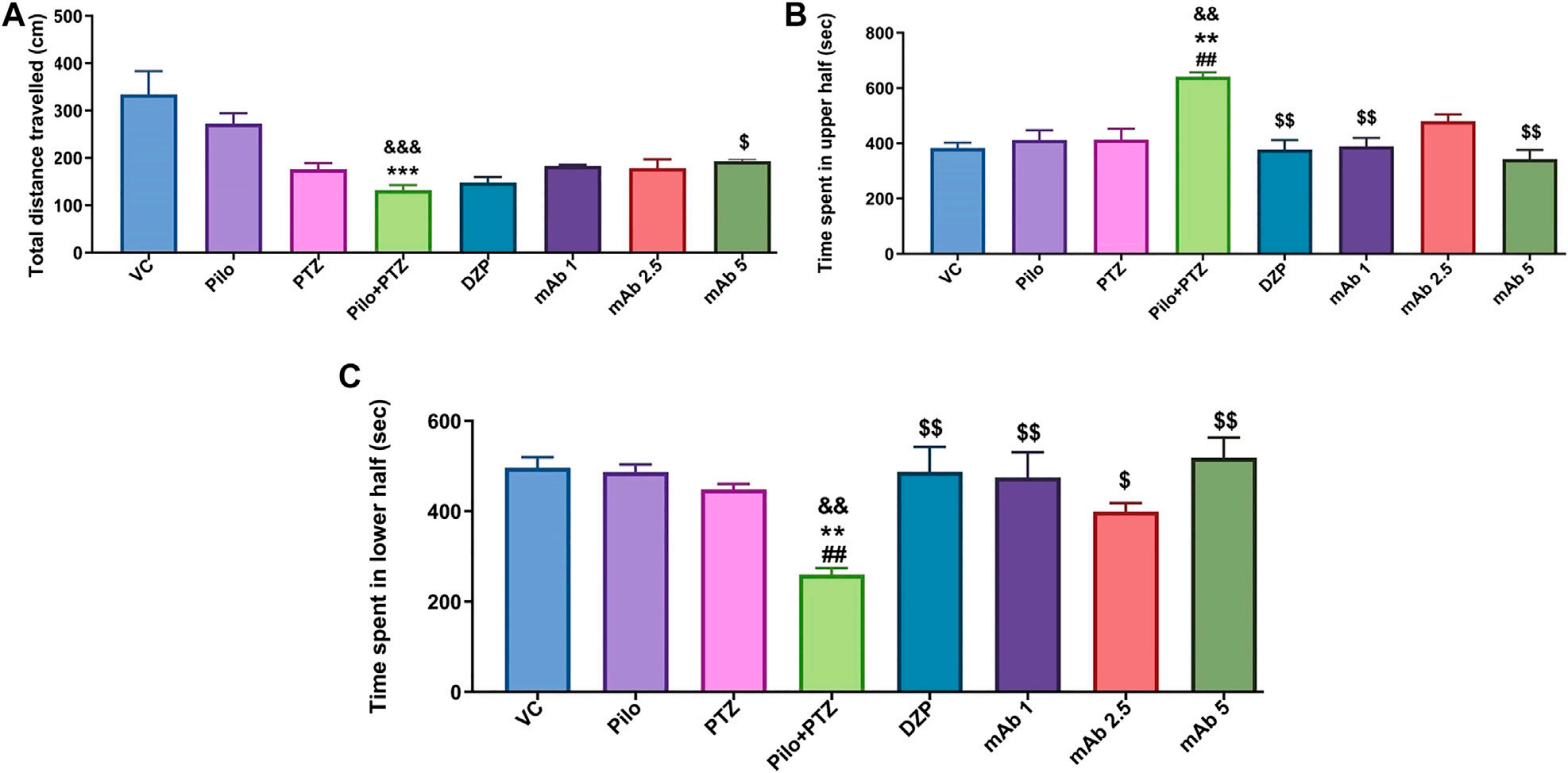
Mean locomotor parameters of the experimental groups (n = 12) at day 10. Mean locomotion parameters of the experimental groups representing the mean total distance traveled (cm) **(A)**, mean time spent in upper half of the tank in (seconds) **(B)** and the mean time spent in lower half of the tank (seconds) **(C)**. Data are represented as mean ± SEM, n = 12, and statistically analyzed by one-way ANOVA followed by Sidak’s multiple comparison test. ^&&&^ *p* < 0.001 and ^&&^ *p* < 0.01 represent the significance level when second hit PTZ group is compared with VC group. ***p < 0.001 and **p < 0.01 represent the significance level when second hit PTZ group is compared with Pilo group. ^$$^ *p* < 0.01 and ^$^ *p* < 0.05 represents the significance level when DZP, mAb 1, mAb 2.5, and mAb 5 treated groups is compared with second hit PTZ group.

Second hit PTZ group demonstrated significant reduction in the total distance traveled when compared to the VC (^&&&^ *p* < 0.001) and Pilo (*** *p* < 0.001) group in day 10 (Figure 4A). There was a non-significant upregulation in the total distance traveled in the experimental groups pre-treated with DZP, mAb 1, mAb 2.5, and mAb 5 whereas significant (^$^ *p* < 0.05) upregulation was observed only with mAb 5 (Figure 4A).

For the parameter of time spend in upper half of the tank, second hit PTZ group demonstrated significant increase when compared with VC (^&&^ *p* < 0.01), Pilo (**p < 0.01), and PTZ (^##^ *p* < 0.01), groups on day 10 (Figure 4B). Second hit PTZ induced upregulation in the time spend in upper half of the tank was significantly decreased upon pre-treatment with DZP (^$$^ *p* < 0.01), mAb 1 (^$$^ *p* < 0.01), and mAb 5 (^$$^ *p* < 0.01) (Figure 4B). However, the decrease in the time spend in upper half of the tank upon pre-treatment with mAb 2.5 was not statistically significant when compared with second hit PTZ group.

The other locomotor parameter is the time spend in the lower of the tank, where second hit PTZ group demonstrated significantly decreased time spend in lower half of the tank when compared with VC (^&&^ *p* < 0.01), Pilo (*** *p* < 0.001), and PTZ (^##^ *p* < 0.01), groups, respectively (Figure 4C). On the contrary, pre-treatment with DZP (^$$^ *p* < 0.01), mAb 1 (^$$^ *p* < 0.01), mAb 2.5 (^$^ *p* < 0.05), and mAb 5 (^$$^ *p* < 0.01), significantly increased the time spend in the lower of the tank when compared with second hit PTZ group (Figure 4C).

Our finding suggests that mAbs ameliorated second hit PTZ induced behavioral impairment as evident by normalized swimming pattern, increase in the total distance traveled, decrease in time spend in upper half of the tank and increase in time spend in lower half of the tank.

### Seizure Analysis

**FIGURE 5:**
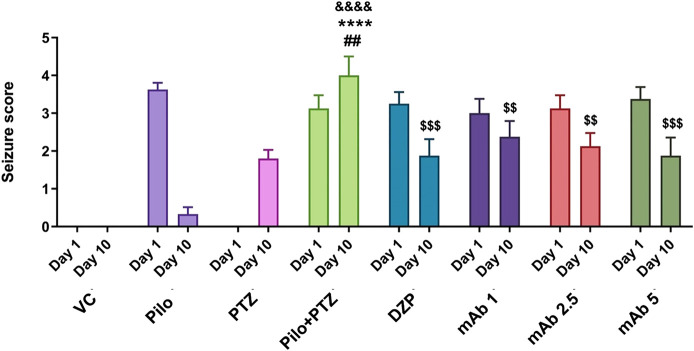
Anti-epileptic effect of mAb against second hit PTZ-induced seizures. Seizure scores of corresponding experimental groups are demonstrated for day 10. Data are represented as mean ± SEM, n = 12, and statistically analyzed by one-way ANOVA followed by Sidak’s multiple comparison test. ^&&&&^ *p* < 0.0001, ****p < 0.0001 and ^##^ *p* < 0.01 represent the significance level when second hit PTZ group is compared with VC group, Pilo group and PTZ group, respectively. ^$$$^ *p* < 0.001 and ^$$^ *p* < 0.01 represents the significance level when DZP, mAb 1, mAb 2.5, and mAb 5 treated groups is compared with second hit PTZ group.

To evaluate the anti-epileptic effect of mAb, we performed a seizure analysis of corresponding experimental groups as quantified by the seizure score. The seizure recording was performed until 15 min and seizure score beyond 15 min was not considered. A maximum seizure score of 0 was assigned to the VC group. On day 10, seizure score was significantly increased in second hit PTZ group when compared with the VC (^&&&&^ *p* < 0.0001), Pilo (**** *p* < 0.0001) and PTZ (^##^ *p* < 0.01) groups, respectively (Figure 5). Precisely, on day 10, second hit PTZ group exhibited full blown seizures of score 4, which was significantly higher when compared to the Pilo and PTZ groups which demonstrated seizure score of 0.3 and 1.8 respectively (Figure 5). The significant difference in the seizure score between the second hit PTZ group; and Pilo and PTZ group is attributed to the fact that seizure score gradually decline in the Pilo group from 3.6 (day 1) to 0.3 (day 10). And same convulsive dose of PTZ which produces full blown seizure in day 10 in second hit PTZ group only produces a seizure score of 1.8 on the PTZ group (day 10) which is due to the fact that second hit PTZ group is prior injected with Pilocarpine on day 1. These difference in the seizure score between VC, Pilo and PTZ groups signifies the validity of second hit PTZ group.

The second hit PTZ induced upregulation in seizure score was significantly downregulated upon pre-treatment with DZP (^$$$^ *p* < 0.001). In addition, pre-treatment with mAb 1 (^$$^ *p* < 0.01), mAb 2.5 (^$$^ *p* < 0.01), and mAb 5 (^$$$^ *p* < 0.001) significantly suppressed the second hit PTZ induced seizure in dose-dependent manner (Figure 4). These findings reflect that mAb demonstrated anti-convulsive effect against second hit PTZ induced seizure. Worth mentioning here is that we do not observe the disease-modifying effects of mAb as pre-treatment with three doses of mAb does not completely halt the seizure progression.

### Learning and Memory Behavior

**FIGURE 6:**
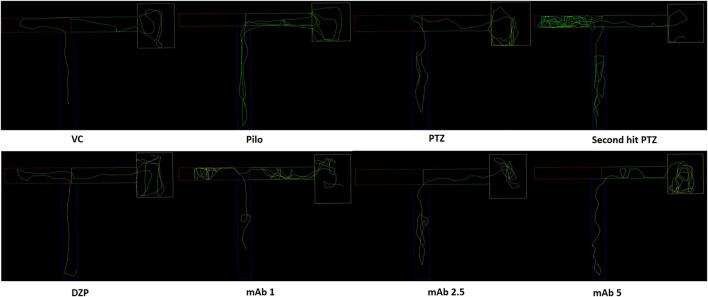
T-maze tracking pattern (24 h trial) of locomotor behavior for the corresponding experimental groups (n = 12). Represents the swimming pattern of VC, Pilo, PTZ, second hit PTZ, DZP, mAb 1, mAb 2.5, and mAb 5 groups.

To evaluate the effect of mAb on second hit PTZ induced cognitive decline, we performed a T-maze test for the corresponding experimental groups at 0, 3 and 24 h interval. The swimming pattern of the fish from VC, Pilo and PTZ groups were found to be normal, with a single right turn to the deepest chamber and minimal entry to the wrong arm (Figure 6) (The second hit PTZ group demonstrated memory impairment as evident by erratic swimming patterns and suboptimal route to the deeper chamber exhibiting frequent back turns into the long and wrong arm (Figure 6). Moreover, second hit PTZ group spend more time in the long and wrong arm of the T-maze before reaching to the favourable environment (deepest chamber) (Figure 6) DZP treated group displayed similar swimming pattern to VC group and reached a deeper chamber, with fewer entries into the wrong arm (Figure 6). Moreover,, mAb 2.5 and mAb 5 ameliorated the second hit PTZ induced memory impairment as evident by straightforward traveling to the deeper chamber without any back turns as well as with no entry into the wrong arm (Figure 6). On the contrary, mAb 1 does not completely ameliorated an impaired memory induced by second hit PTZ as evidenced by the fewer entries into the wrong arm before reaching the favourable chamber.

**FIGURE 7:**
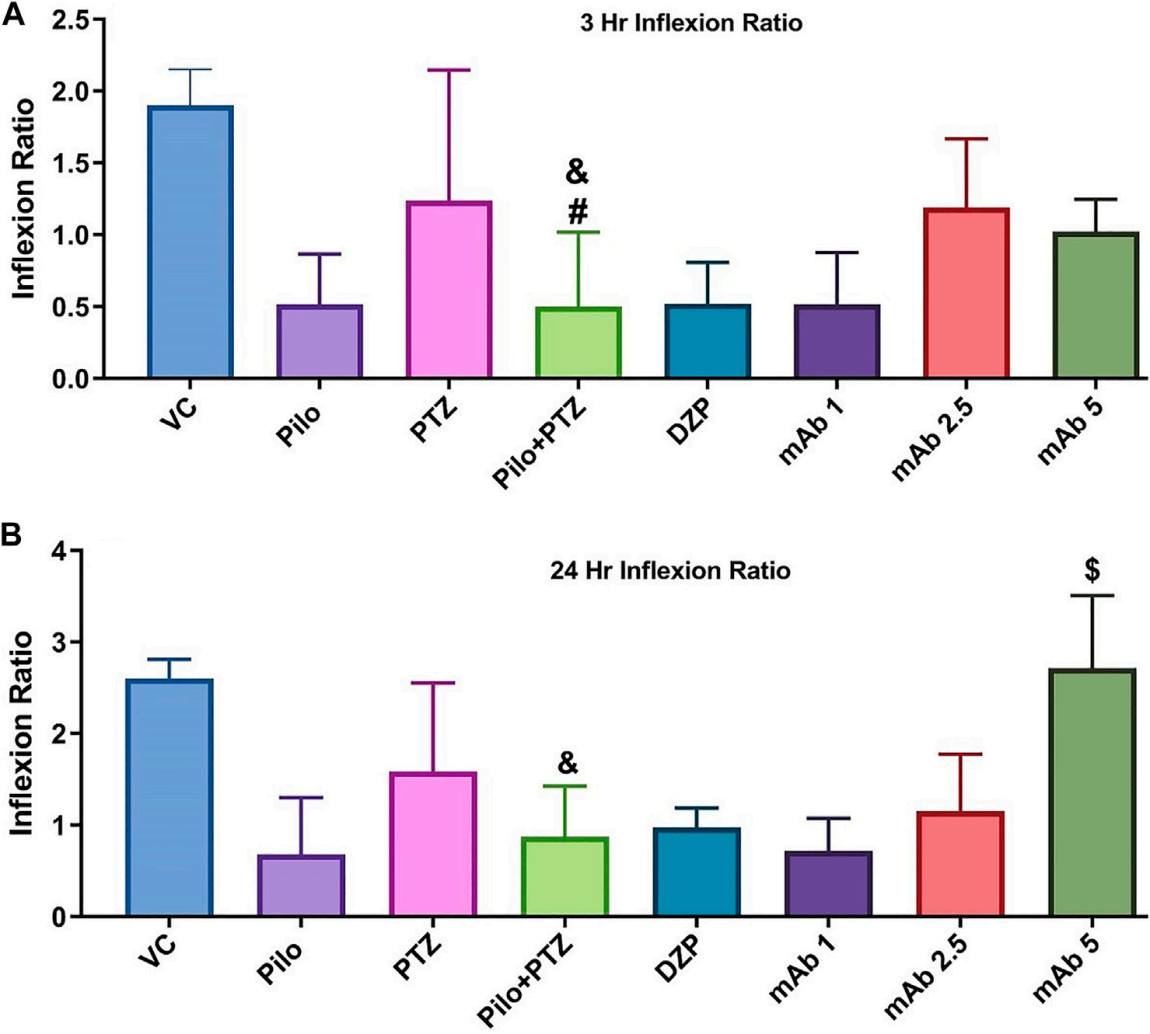
Graph plot of the inflection ratio at the 3 h and 24 h T-maze trial of the corresponding experimental groups. Inflection ratio at the 3 h **(A)** and 24 h **(B)** T-maze trial of the corresponding experimental groups Data was represented as mean ± SEM, n = 12, and statistically analyzed by one-way ANOVA followed by Sidak’s multiple comparison test. ^&^ *p* < 0.05 and ^#^ *p* < 0.05 represents the significance level when second hit PTZ group is compared with VC group and PTZ group respectively. ^$^ *p* < 0.05 represents the significance level when DZP, mAb 1, mAb 2.5, and mAb 5 treated groups is compared with second hit PTZ group.

Upon assessing the memory function in the T-maze test, second hit PTZ group demonstrated significantly depleted memory as evidenced by the reduction in inflection ration at 3 h when compared to VC (^&^ *p* < 0.05), and PTZ (^#^ *p* < 0.05), group, respectively (Figure 7). Similar impairment was obtained at 24 h trial in second hit PTZ group when compared to the VC group (^&^ *p* < 0.05). Pre-treatment with mAb 1, mAb 2.5, and mAb 5 ameliorated the second hit PTZ induced memory impairment as evidenced by increased inflection ration at 3 h and 24 h (Figure 7). However, the results were statistically significant (^$^ *p* < 0.05), with higher dose i.e. mAb 5. Nevertheless, our finding reflects that mAbs rescued against second hit PTZ induced memory impairment, however, the dose-responsive effects were not observed.

### Neurotransmitters Levels

**FIGURE 8:**
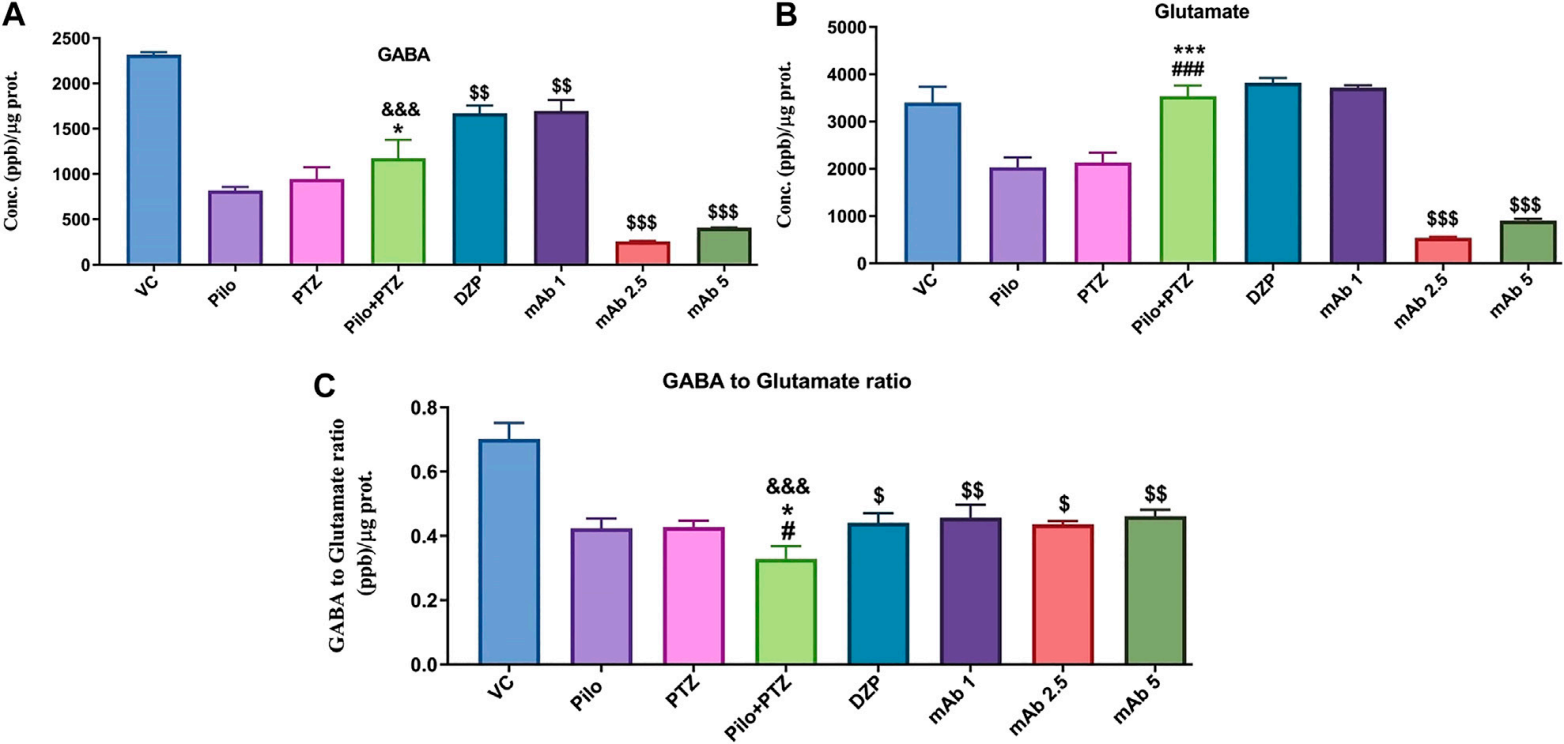
Analysis of Neurotransmitters level in epileptic zebrafish brains after pre-treatment with anti-HMGB1 mAb. Concentration of GABA **(A)** and Glutamate **(B)** and GABA to Glutamate ratio **(C)** in the corresponding experimental groups. Data are represented as mean ± SEM, n = 6, and statistically analyzed by one-way ANOVA followed by Sidak’s multiple comparison test. ^&&&^ *p* < 0.001 represents the significance level when second hit PTZ group is compared with VC group. ***p < 0.001 and *p < 0.05 represents the significance level when second hit PTZ group is compared with Pilo group. ^###^ *p* < 0.001 and ^#^ *p* < 0.05 represents the significance level when second hit PTZ group is compared with PTZ group. ^$$$^ *p* < 0.001, ^$$^ *p* < 0.01 and ^$^ *p* < 0.05 represent the significance level when DZP, mAb 1, mAb 2.5, and mAb 5 treated groups is compared with second hit PTZ group.

The modulation of neurotransmitters underlies the brain response to the neuronal firings during epileptic seizures. To investigate the modulation of neurotransmitters upon anti-HMGB1 mAb treatment we quantified the concentrations of GABA and Glutamate via LC-MS/MS. There was a significant downregulation in the levels of GABA in the second hit PTZ group as compared to VC group (^&&&^ *p* < 0.001) (Figure 8A). Moreover, when compared to the Pilo group, there is upregulation in the GABA level in second hit PTZ group (*p < 0.05). Second hit PTZ induced reduction in GABA level were significantly upregulated upon pre-treatment with DZP (^$$^ *p* < 0.01) and mAb 1 (^$$^ *p* < 0.01). However, there was a significant decrement in the GABA level in the groups treated with mAb 2.5 (^$$$^ *p* < 0.001) and mAb 5 (^$$$^ *p* < 0.001) (Figure 8A).

Glutamate level was significantly upregulated in second hit PTZ group when compared with Pilo (^***^ *p* < 0.001), and PTZ (^###^ *p* < 0.001) group whereas no significant modulation was there when compared with VC group (Figure 8B). Pre-treatment with anti-HMGB1 mAb protects against second hit PTZ induced seizures as evident by the decreased level of Glutamate mainly in the mAb 2.5 (^$$$^ *p* < 0.001) and mAb 5 (^$$$^ *p* < 0.001) groups (Figure 8B). However, no modulation in GABA level was there on DZP and mAb 1 groups respectively.

Second hit PTZ group demonstrated significant reduction in the GABA/Glutamate ratio when compared to VC (^&&&^ *p* < 0.001), Pilo (*p < 0.05) and PTZ (^#^ *p* < 0.05) groups (Figure 8C). This reflects the possible disruption in the balance of excitatory and inhibitory mechanism in the second hit PTZ group. On the contrary, second hit PTZ induced decrease in GABA/Glutamate was increased significantly in DZP (^$^ *p* < 0.05), mAb 1 (^$$^ *p* < 0.01), mAb 2. 5 (^$^ *p* < 0.05), and mAb 5 (^$$^ *p* < 0.01), respectively (Figure 8C). The increase in GABA/Glutamate ration reflect an amelioration of second hit PTZ induced imbalance in excitatory and inhibitory mechanism.

### HMGB1, TLR4, NF-κB, and TNF-α mRNA Expression

Though the etiopathogenesis of seizure generation is still obscure, there is an increased understanding about the role of inflammatory mediators in generation of epileptic seizures. We have assessed the mRNA expression level of four inflammatory markers (HMGB1, TLR4, NF-κB, and TNF-α) upon mAb treatment to evaluate the anti-inflammatory mechanism of mAb.

**FIGURE 9:**
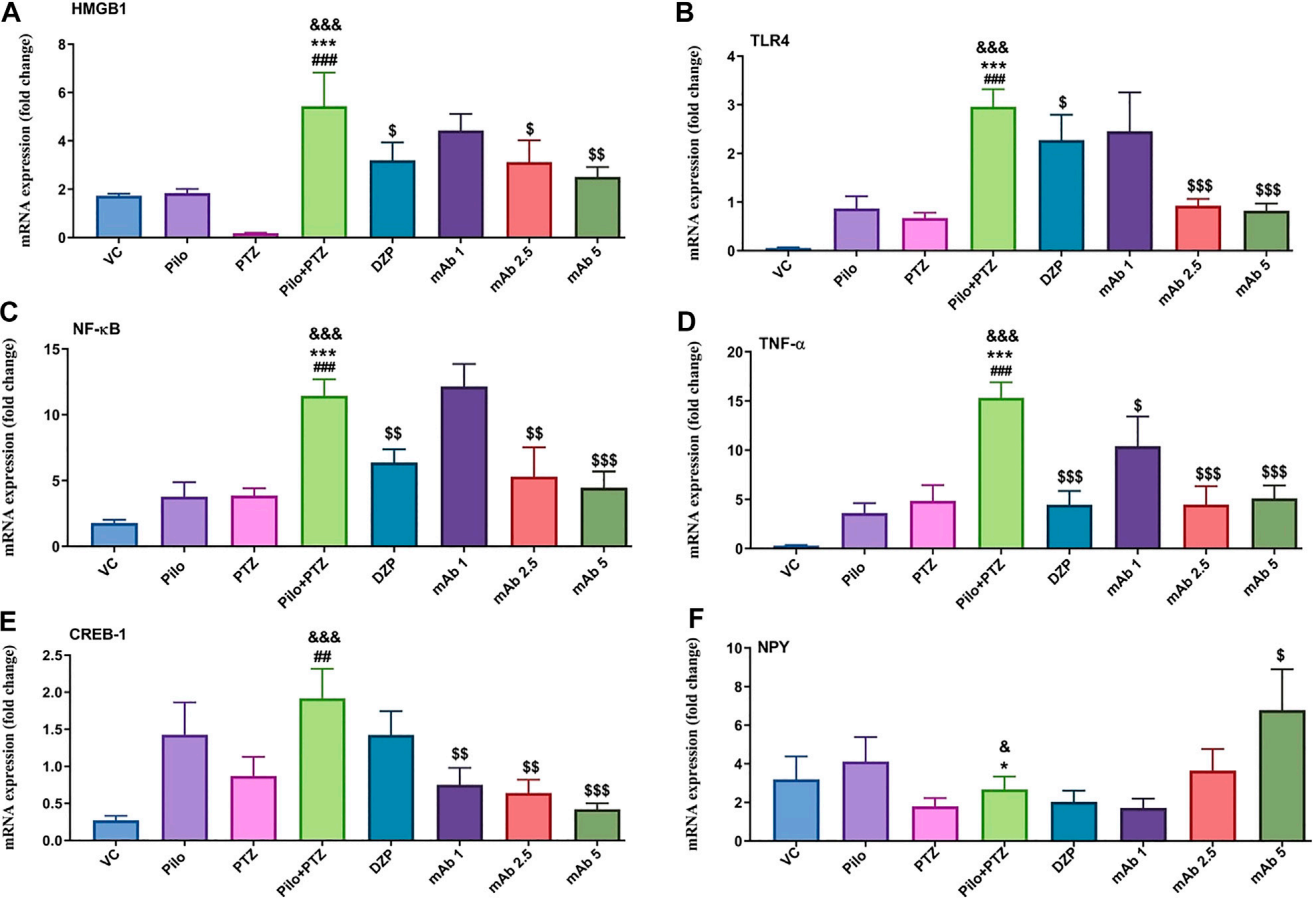
Modulation of inflammatory mediator (HMGB1, TLR4, NF-κB, TNF-α), transcription factor (CREB-1) and neuropeptide (NPY). The graph plot is represented as a fold changes in mRNA expression of HMGB1 **(A)**, TLR4 **(B)**, NF-κB **(C)**, TNF-α **(D)**, CREB-1 **(E)** and NPY **(F)**. Data are represented as mean ± SEM, n = 6, and statistically analyzed by one-way ANOVA followed by Sidak’s multiple comparison test. ^&&&^ *p* < 0.001 and ^&^ *p* < 0.05 represents the significance level when second hit PTZ group is compared with VC group. ***p < 0.001 and *p < 0.05 represents the significance level when second hit PTZ group is compared with Pilo group. ^###^ *p* < 0.001 and ^##^ *p* < 0.01 represents the significance level when second hit PTZ group is compared with PTZ group. ^$$$^ *p* < 0.001, ^$$^ *p* < 0.01 and ^$^ *p* < 0.05 represent the significance level when DZP, mAb 1, mAb 2.5, and mAb 5 treated groups is compared with second hit PTZ group.

Second hit PTZ resulted in significant upregulation of mRNA expression levels of HMGB1 and TLR4 when compared with VC (^&&&^ *p* < 0.001), Pilo (^***^ *p* < 0.001), and PTZ (^###^ *p* < 0.001) group respectively (Figures 9A,B). On the contrary, the increased upregulation in mRNA expression level of HMGB1 and TLR4 was downregulated upon pre-treatment with DZP and anti-HMGB1 mAbs (Figures 9A,B). DZP group demonstrated significant downregulation (^$^ *p* < 0.05) in mRNA expression of HMGB1 and TLR4 when compared to the second hit PTZ group. Pre-treatment with mAb 2.5 (^$^ *p* < 0.05), and mAb 5 (^$$^ *p* < 0.01) reduced mRNA expression of HMGB1 (Figure 9A). Moreover, significant reduction in TLR4 expression was reported up on pre-treatment with mAb 2.5 (^$$$^ *p* < 0.001) and mAb 5 (^$$$^ *p* < 0.001) (Figure 9B). However, the decrease in the mRNA level of HMGB1 and TLR4 by mAb 1 was not statistically significant (Figures 9A,B).

There was a significant increment in the mRNA expression level of NF-κB and TNF-α in the second hit PTZ group in comparison with VC (^&&&^ *p* < 0.001), Pilo (^***^ *p* < 0.001), and PTZ (^###^ *p* < 0.001) group respectively (Figures 9C,D). On the contrary, pre-treatment with DZP (^$$^ *p* < 0.01), mAb 2. 5 (^$$^ *p* < 0.01), and mAb 5 (^$$$^ *p* < 0.001) resulted in the decrease of NF-κB level (Figure 9C). Similar decrement in the mRNA expression level of TNF-α was observed in DZP (^$$$^ *p* < 0.001), mAb 1 (^$^ *p* < 0.05), mAb 2.5 (^$$$^ *p* < 0.001), and mAb 5 (^$$$^ *p* < 0.001) groups respectively.

### mRNA Expression of Transcription Factor (CREB-1), and Neuropeptide (NPY)

To evaluate either anti-HMGB1 mAb treatment modulates the transcription factor and neuropeptides having role in learning and memory, and neuronal plasticity we have assessed the mRNA expression level of CREB-1 and NPY.

Second hit PTZ group showed significant increment in the CREB-1 mRNA level when compared with VC (^&&&^ *p* < 0.001), and PTZ (^##^ *p* < 0.01) group respectively (Figure 9E). Non-significant downregulation in the mRNA expression of CREB-1 was observed in the DZP group when compared to the second hit PTZ group (Figure 9E). Moreover, a dose-dependent decrease in the mRNA expression level of CREB-1 was observed in mAb 1 (^$$^ *p* < 0.01), mAb 2.5 (^$$^ *p* < 0.01), and mAb 5 (^$$$^ *p* < 0.001) pre-treated groups respectively (Figure 9E).

There was a significant downregulation in the mRNA expression of NPY in the second hit PTZ group when compared to the VC (^&^ *p* < 0.05), and Pilo (^*^ *p* < 0.05) groups respectively (Figure 9F). There was a non-significant decrease in the mRNA expression of NPY in groups pre-treated with DZP and mAb 1 when compared with the second hit PTZ group. However, pre-treatment with mAb 5 significantly (^$^ *p* < 0.05) upregulated the expression of NPY when compared to the second hit PTZ group (Figure 9F).

## Discussion

Despite recent advances in basic and clinical research, the etiopathology of seizure generation is not completely understood and lacks the effective disease modifying AEDs. In the quest of exploring potential therapeutic strategy against epilepsy, HMGB1 has emerged as an promising target against epilepsy (Fu et al., 2017; Zhao et al., 2017; Li et al., 2019). However, the anti-convulsant effect of mAb and ameliorating effect on learning and memory has not been yet investigated on seizure model in adult zebrafish. Herein, we reported that mAb reduced second hit PTZ-induced seizures and ameliorated learning and memory and downregulated the inflammatory mediators.

### Second Hit PTZ Induced Seizure as Novel Seizure Model in Adult Zebrafish

The mainstream pre-clinical research related to epileptogenesis are carried out utilizing rodents as well as from human tissues obtained during surgical resection (Maroso et al., 2010; Roseti et al., 2015). Nevertheless, zebrafish also known as *Danio rerio* (Cyprinidae family) (Jagdale et al., 2020) has gained increased attention as a promising *in vivo* animal model for diverse ranges of neurological diseases including epilepsy (Baraban et al., 2005; Mussulini et al., 2013; Kundap et al., 2017; Mussulini et al., 2018). Zebrafish can be utilized to investigate the pathogenesis of epilepsy, screening of anti-epileptic drugs, validation of target and most importantly can be modeled into novel seizure model recapitulating the clinical phenotype. Compared to rodents seizure model, zebrafish seizure model possesses a benefits of minimal drugs requirement, shorter duration of assays, resemblance and easy quantification of seizure-like behavior, possibility of recording abnormal neuronal discharge by electroencephalography (EEG) and its transparent nature (Buenafe et al., 2013; Pisera-Fuster et al., 2017; Zhang et al., 2020). Despite the availability of Kainic acid (Mussulini et al., 2018), PTZ (Mussulini et al., 2013) and Pilocarpine (Paudel et al., 2020e) induced seizure model in adult zebrafish, no earlier study has reported a longitudinal (10 days) chronic seizure model in adult zebrafish.

The current study modeled a second hit PTZ induced seizure model in adult zebrafish. On day 10, compared to the VC, Pilo and PTZ groups, second hit PTZ group depicted full blown seizures of score 4 The seizure score in Pilo group decreased gradually with time whereas in PTZ group, the same sub-convulsive dose of PTZ does not produce the comparable seizure score as in the second hit PTZ group. This is attributed to the fact that, second hit PTZ group is prior stimulated with Pilocarpine in day 1 whereas in PTZ group, zebrafish were only injected with VC in day 1 rather than The difference in the seizure score among the Pilo and PTZ group justifies the validity of second hit PTZ seizure model. Worth mentioning here is that, only in second hit PTZ group, the animals are in the seizure stage from day 1 to day 10 when compared to VC, Pilo, and PTZ groups Hence, the second hit PTZ induced seizure model developed in the current study offers a benefit for long-term investigation which are currently not offered by the other seizure model in adult zebrafish induced by PTZ (Mussulini et al., 2013) and Kainic acid (Mussulini et al., 2018).

### mAb Ameliorates Second Hit PTZ Induced Behavioral Impairment

Several indicators such as abnormal locomotor pattern and EEG recordings, seizure like phenotypes, increased neuronal activity etc improves the accuracy of seizure recognition in zebrafish seizure model (Afrikanova et al., 2013; Jin et al., 2020; Zhang et al., 2020). Worth mentioning here is that adult zebrafish exhibited different behavioral response post-seizure depending upon the seizure model induced by PTZ (Mussulini et al., 2013), Kainic acid (Mussulini et al., 2018) and Pilocarpine (Paudel et al., 2020e). This difference might be due to the different mechanism of action of the pro-convulsants and difference in the quantification of seizure score among the seizure models.

Second hit PTZ group demonstrated abnormal swimming pattern with swimming mainly in a preferred region of the tank and decrease in the total distance traveled throughout the tank. Pre-treatment with mAb 1, mAb 2.5, and mAb 5 normalized the abnormal swimming pattern as evident by the swimming pattern of zebrafish throughout the tank subsequently leading to the increase in total distance traveled. This might be plausibly due to the anti-seizure effect of mAb leading the zebrafish to move throughout the tank after recovering from second hit PTZ induced seizure.

The increase and decrease in the time spend by the zebrafish in the upper half and lower half of the tank respectively in the second hit PTZ group is different from the earlier reported PTZ induced seizure model in adult zebrafish (Kundap et al., 2017). Worth mentioning here is that the variation might be due to the difference in the seizure model and execution of experimental protocol between the study and possibly by other factors that could be unraveled upon future studies. However, mAb 1, mAb 2.5, and mAb 5 reversed the second hit PTZ induced increase in and decrease in the time spend by the zebrafish in the upper half and lower half of the tank, respectively. All these findings reflect that mAb ameliorates second hit PTZ induced behavioral impairments.

### Anti-convulsant Effect of mAb Against Second Hit PTZ Induced Seizure

HMGB1 has emerged as a therapeutic target against arrays of inflammation mediated pathologies (Andersson et al., 2018). In a rodent model of epilepsy, targeting/inhibiting HMGB1 by BoxA (fragment of HMGB1 with antagonistic activity) and other HMGB1 neutralization strategies such as anti-HMGB1 mAb and Glycyrrhizin demonstrated reduction in frequency and duration of seizure (Maroso et al., 2010), disease modifying anti-epileptogenic effects (Zhao et al., 2017) and neuroprotective effects (Li et al., 2019) respectively. This promising findings upon HMGB1 neutralization against epileptic seizure has prompted to further evaluate the anti-epileptic effect of mAb in zebrafish seizure model.

We observed that pre-treatment with mAb 1, mAb 2.5, and mAb 5 dose-dependently reduces second hit PTZ induced seizure as evident by the reduced seizure score when compared to the second hit PTZ group. This finding reflects the anti-convulsive effect of anti-HMGB1 mAb. However, we did not observe a compelling disease-modifying anti-epileptic effects of anti-HMGB1 mAb, as observed in a rodent model of epilepsy (Fu et al., 2017; Zhao et al., 2017). This disparity in the result might be attributed to the fact that the current study employed a single dosing pattern of mAbs, which might not be able to completely halt the seizure progression. In addition, difference in the treatment approach i.e. pre-treatment and post-treatment with mAb might play a role (Ying et al., 2020).

### Effect of mAb Against Second Hit PTZ Induced Memory Impairment

Several lines of investigation have earlier reported that epileptic zebrafish exhibited memory impairment (Kundap et al., 2017; Kundap et al., 2019a) which is in agreement with the fact that memory impairment is associated with epilepsy (Kesselmayer et al., 2019; Laurent et al., 2020). This reflects that the future therapeutic strategies against epileptic seizures should not only exert the disease supression but also ameliorate the related memory impairment. Despite the reported anti-epileptic effect of mAb against epileptic seizures in rodent models, the ameliorating effect of mAb against epilepsy related memory impairment is not yet explored in zebrafish seizure model. The reduced inflection ratio in the second hit PTZ group at 3 and 24 h when compared to the VC group reflect the memory impairment in the second hit PTZ group. This finding is in agreement with earlier finding reporting PTZ induced cognitive impairment in adult zebrafish (Kundap et al., 2019a). These impairment in memory induced by second hit PTZ was ameliorated upon treatment with mAbs as evidenced by increased inflection ratio at 3 h and 24 h. Worth mentioning here is that, the significant upregulation of inflection ratio was only observed with mAb 5 at 24 h T-maze test. This might be possibly due to the fact that pre-treatment with mAb 1, mAb 2.5, and mAb 5 (single dose) only at day 10 may not exert therapeutic effect to rescue the second hit PTZ induced memory impairment. Nevertheless, our finding suggests that mAb might be a plausible therapeutic strategy against epilepsy related memory impairment. However, future studies investigating the memory ameliorating effect of mAb with varied dose will increase our current understanding regarding mAb effects against second hit PTZ induced memory impairment.

### mAb Effects on GABA, Glutamate, and Ratio of GABA/Glutamate

GABA contribute a significance role in the learning and memory (Luo et al., 2011) and is the primary neuroinhibitory in the CNS. GABA_A_ receptors signaling exhibits diverse context-specific activities that can either prevent or promote epileptogenesis and seizure generation (Kaila et al., 2014). Reflecting the disturbance in the GABAergic system, we demonstrated a decreased level of GABA in the second hit PTZ group corroborating the similar finding in PTZ induced seizure model in adult zebrafish (Choo et al., 2019). Pre-treatment with mAb 2.5 and mAb 5 decreases the GABA level when compared to the second hit PTZ group. This might be due to the fact that DZP being positive allosteric modulator of the GABA_A_ receptors, HMGB1 decrease the effectiveness of GABA-mediated inhibitory functions (Zhao et al., 2019).

Seizures are acknowledged as a result of an imbalance among excitatory and inhibitory neuronal activities. Enhanced Glutamate release into the synaptic clefts, in part, might leads to excitotoxic lesion-induced epilepsy and enhanced Glutamate concentration that released from hyperexcitation of the neuronal network, might play a key role in the initiation and spread of seizures (Kaneko et al., 2017). Glutamate is an excitatory amino acid that has been implicated in the pathogenesis of epilepsy (Miller et al., 1997; Chapman, 2000). Glutamate release may lead to an intracellular calcium ultimately leading to cell death (Holmes, 2002), as well as Glutamate toxicity might impair learning and memory (Izquierdo and Medina, 1997). The increased concentration of Glutamate in the second hit PTZ group of our study is in similar line to the PTZ induced seizure model in adult zebrafish (Kundap et al., 2017) speculating possibility of cell death and impaired learning and memory in the second hit PTZ group. Pre-treatment with mAb 2.5, and mAb 5reduces the Glutamate level that may reflect suppression of seizures, reduction in cell death and improved learning and memory. Our findings corroborate with earlier findings where treatment with HMGB1 (1 µg/ml) to primary rat neuronal cells (PRNCs) downregulates the expression levels of Glutamate metabolism associated enzymes Glutamate decarboxylase67 (GAD67) and Glutamate dehydrogenase (GLUD1/2) (Kaneko et al., 2017). However, difference in the modulation of Glutamate by extracellular HMGB1 in difference type of seizure models and study types (*in vitro* and *in vivo*) is not yet known. The modulation of GABA by different dose of mAb is in different line from the current understanding where amelioration of seizure leads to the upregulated level of GABA in seizure model in adult zebrafish (Kundap et al., 2019a). Hence, further studies are warranted to confirm our finding, that could explore on the modulation of GABA by mAb HMGB1 and GABA.

PTZ being acknowledge to act via blocking the GABA_A_ receptor, resulted in the reduced ration of GABA to Glutamate in the second hit PTZ group, which might be attributed to the fact that PTZ induces the GABA inhibition and upregulates the Glutamate excitatory activity. Our finding corroborates the earlier study reporting reduced GABA to Glutamate ratio upon PTZ administration in adult seizure model (Chung et al., 2020). On the contrary, pre-treatment with mAb 1, mAb 2.5, and mAb 5 increased concentration of GABA to Glutamate ratio resembling the balance in excitatory and inhibitory neurotransmission which might be due to the suppressed seizure upon mAb pre-treatment. Similar increment in the GABA and Glutamate ratio has been observed upon recovery from PTZ induced seizure in adult zebrafish (Chung et al., 2020).

### Second Hit PTZ Induced Inflammatory Response Is Alleviated by mAb

Neuroinflammation and changes in the levels of related pro-inflammatory cytokines has been acknowledged to cause long-term alterations in the brain (Amini et al., 2018). Plethora of pre-clinical and clinical studies has reported the presence and upregulation of inflammatory mediators (the brain, cerebrospinal fluid, and blood) in epileptic conditions (Vezzani and Granata, 2005). Though the complex etiopathogenesis of epilepsy still remained elusive, there is an increased understanding on the contribution of neuroinflammation in generation and perpetuation of epileptic seizures (Aronica et al., 2017; Vezzani et al., 2019; Hodges and Lugo, 2020).

HMGB1 being a pro-inflammatory like cytokine and its role in neuroinflammation (Ravizza et al., 2018; Paudel et al., 2019a) has gained significant attention in epilepsy (Maroso et al., 2010; Paudel et al., 2018a). Increased upregulation and expression of HMGB1 in animal models and human temporal lobe epilepsy (TLE) and modification of epileptic seizures upon treatment with antagonist of HMGB1 strengthen the role of HMGB1 in generation of epileptic seizure (Maroso et al., 2010). Upregulated mRNA expression of HMGB1 has been observed in a Pilocarpine induced epilepsy model in mice (Ying et al., 2020) and in seizure model in adult zebrafish (Paudel et al., 2020d). In a similar line with earlier reported finding, our study corroborated the increased mRNA expression of HMGB1 in a second hit PTZ group. This supports the notion that HMGB1 might play a possible role in generating seizures in the second hit PTZ group. However, pre-treatment with mAb 1, mAb 2.5, and mAb 5 reduces the mRNA expression of HMGB1 that might be possibly due to interfering with the release and expression of HMGB1.

The contribution of HMGB1 in seizure generation is mainly facilitated by interaction with TLR4 and RAGE. NF-κB is a key nuclear transcription factor crucial for an innate and adaptive immunity. There is an activation of the TLR4/NF-κB signaling axis in epilepsy as evident by an upregulated expression of TLR4 and NF-κB in epileptic animals and inhibiting the TLR4 and NF-κB represent a promising strategy against epileptic seizure (Liu et al., 2017; Shi et al., 2018; Qu et al., 2019). Herein, we observed an upregulated mRNA expression of TLR4 and NF-κB in the second hit PTZ group. Our findings are in corroboration with earlier findings from rodents and clinical experimentation where TLR4 (Fu et al., 2017; Amini et al., 2018; Shi et al., 2018) and NF-κB level (Choo et al., 2018) has been reported to be upregulated in epileptic condition. We hypothesize an activation of the HMGB1-TLR4-NF-κB signaling pathway as evident by an upregulated mRNA expression level of HMGB1, TLR4, and NF-κB in second hit PTZ seizure model. Moreover, we observed a downregulation/inhibition in the mRNA expression of HMGB1, TLR4, and NF-κB upon pre-treatment with mAb 1, mAb 2.5, and mAb 5 speculating the supression of second hit PTZ induced activation of HMGB1-TLR4-NF-κB signaling axis. HMGB1-TLR4/NF-κB interaction might play a crucial role in facilitating the neuroprotective effects in the experimental models of seizures, suggesting that overexpression of HMGB1 might activates TLR4/NF-κB signaling pathway, and might be beneficial for the treatment of neurological disorders including epilepsy (Maroso et al., 2010; Wang et al., 2010).

TNF-α is a pro-inflammatory cytokine that acts mainly on two types of receptors namely p55 (TNF-α R1) and p75 (TNF-α R2) receptors (Balosso et al., 2009). Endogenous brain levels and the receptor subtypes of TNF-α are the crucial aspects determining the effect of TNF-α on seizures (Vezzani and Granata, 2005). TNF-α acts as an anticonvulsant probably via interacting with the neuronal p75 receptors when injected in the hippocampus of the kainate induced seizures. On the contrary, mice with p55 receptor knock out exhibited decreased susceptibility of seizures (Balosso et al., 2005). However, upregulated expression of TNF-α is evident in the epileptic conditions either in rodents (Ashhab et al., 2013) or in adult zebrafish (Paudel et al., 2020e). Our finding corroborated the earlier one and reported an upregulated mRNA expression of TNF-α in the second hit PTZ group that was in turn downregulated upon pre-treatment with mAb 1, mAb 2.5, and mAb 5.

Our finding suggests that anti-HMGB1 mAb possess an anti-inflammatory potential as evident by reduction in the second hit PTZ induced upregulated expression of HMGB1, TLR4, NF-κB and TNF-α. This anti-inflammatory effect of anti-HMGB1 mAb might be attributed to the anti-convulsive effect of anti-HMGB1 mAb which suppressed second hit PTZ induced seizure which in turn reduced the neuroinflammation.

### Anti-HMGB1 mAb Rescued Second Hit PTZ Induced Modulation in CREB-1 and NPY

CREB is a transcription factor with established role in plasticity (Lonze and Ginty, 2002) and alteration in the level of CREB has been implicated in several psychiatric diseases (Carlezon et al., 2005). In addition to that, CREB has been well acknowledged for its role in memory formation, neuronal plasticity and long-term memory formation and regulates the genes (BDNF, C-FOS) that are crucial for memory formation (Xing et al., 2019). The increased mRNA expression of CREB-1 in the second hit PTZ group reflects the plausible role of CREB-1 in epilepsy as well as suggest that memory is impaired upon PTZ administration. Acknowledging the role of CREB in TLE associated cognitive decline (Xing et al., 2019), we hypothesize that CREB-1 plausibly contribute to the memory impairment in the second hit PTZ group as evidenced by an increased expression.

NPY is reported as a potent anti-convulsant peptides (Noe et al., 2010). NPY is a 36-amino acid peptides and is among the most studied neuropeptides in epilepsy (Tekgul et al., 2020). An increased NPY expression in brain regions is crucial for learning and memory together with its neuromodulatory and neurotrophic effects implicating a regulatory role for NPY in memory processes (Gøtzsche and Woldbye, 2016). Herein, we observed decreased mRNA expression of NPY in the second hit PTZ group implicating the possibilities of learning and memory abnormalities in an epileptic group. The decreased expression of CREB-1 and upregulated expression of NPY by mAb 1, mAb 2.5, and mAb 5 group reflects the amelioration of second hit PTZ induced memory impairment.

Summing up, we developed a second hit PTZ induced seizure in adult zebrafish that recapitulates the behavioral alterations, memory impairment, modulation/upregulation of inflammatory mediators and several levels of neurotransmitters that are impaired during epilepsy. Furthermore, mAb 1, mAb 2.5, and mAb 5 suppress second hit PTZ induced seizure and related memory impairment which might be due to an anti-inflammatory associated mechanism of mAb via the modulation of HMGB1/TLR4/NF-κB axis. On a limiting aspect, implication of our finding would have been more robust if the epileptic seizures in adult zebrafish has been recorded with EEG and modulation of HMGB1 and TLR4 in protein level has been demonstrated. Nevertheless, our findings offer a novel perspective representing HMGB1 a promising therapeutic target against epilepsy that not only suppress the epileptic seizure but also alleviate the seizure related memory impairment. Our finding suggests.

## Data Availability

The raw data supporting the conclusions of this article will be made available by the authors, without undue reservation.

## Abbreviations

AEDs, Anti-epileptic drugs; CNS, Central nervous system; CREB-1, cAMP response element-binding protein; DZP, Diazepam; GABA, γ-aminobutyric acid; HMGB1, High mobility group box 1; mAb, Anti-HMGB1 monoclonal antibody; mAb, Anti-HMGB1 monoclonal antibody; NF-κB, Nuclear Factor kappa-light-chain-enhancer of activated B cells; NPY, Neuropeptide Y; PTZ, Pentylenetetrazol; TLR4, Toll-like receptor-4; TNF-α, Tumor necrosis factor-α.
